# Pneumatosis Cystoides Intestinalis Discovered During Exploratory Laparotomy

**DOI:** 10.7759/cureus.56032

**Published:** 2024-03-12

**Authors:** Regen K Morgan, Austin Wynn, Mary K Hanisee

**Affiliations:** 1 Archbold Department of Surgery, Alabama College of Osteopathic Medicine, Dothan, USA; 2 Archbold Department of Surgery, Archbold Memorial Hospital, Thomasville, USA

**Keywords:** small bowel pneumatosis, rare cause of acute abdominal pain, exploratory laporotomy, ibs (irritable bowel syndrome), pneumatosis cystoides intestinalis

## Abstract

Pneumatosis cystoides intestinalis (PCI) is a rare condition characterized by gas-filled cysts that develop in the mucosal and submucosal layers of the digestive tract. PCI is typically a benign disease but complications can occur that require surgical intervention. This patient presented to the emergency department with a two-day history of abdominal and back pain. A contrast-enhanced computed tomography (CT) scan of the abdomen and pelvis led to suspicion of free intraperitoneal air potentially due to bowel perforation, and exploratory laparotomy was indicated. Bowel perforation was ruled out during the procedure, but outpouchings of air-filled cysts were found throughout the small bowel's external surface, leading to the diagnosis of PCI. Distinguishing free intraperitoneal air from PCI on imaging presents a challenge to clinicians. Contrast-enhanced abdominal CT is the preferred imaging modality, but PCI remains difficult to diagnose on imaging alone. Spreading awareness of the typical benign nature of PCI alongside the common presentation on imaging may lead to earlier detection, better treatment outcomes, and prevention of unnecessary surgical intervention.

## Introduction

Pneumatosis cystoides intestinalis (PCI) is a rare condition characterized by gas-filled cysts that develop in the mucosal and submucosal layers of the digestive tract [1,2]. The distribution of PCI ranges from the esophagus to the rectum, although the most common location is distal to the splenic flexure. Patients often present with nonspecific abdominal symptoms, and the current standard of diagnostic imaging is contrast-enhanced computed tomography (CT) [2,3]. The prevalence of PCI is estimated to be 0.03% of the population, but this is likely an underestimate due to the asymptomatic nature of most cases [4]. There are several hypotheses on PCI pathogenesis but many knowledge gaps remain regarding diagnosis, prevention, complications, and management.

We present a case of PCI discovered incidentally during an exploratory laparotomy. Contrast-enhanced CT suggested the presence of free intraperitoneal air, while surgical intervention revealed diffuse air-filled cysts covering the small bowel. Our goal is to contribute to PCI research as the discovery of the disease increases while reviewing the current literature regarding pathogenesis and management.

## Case presentation

**Figure 1:**
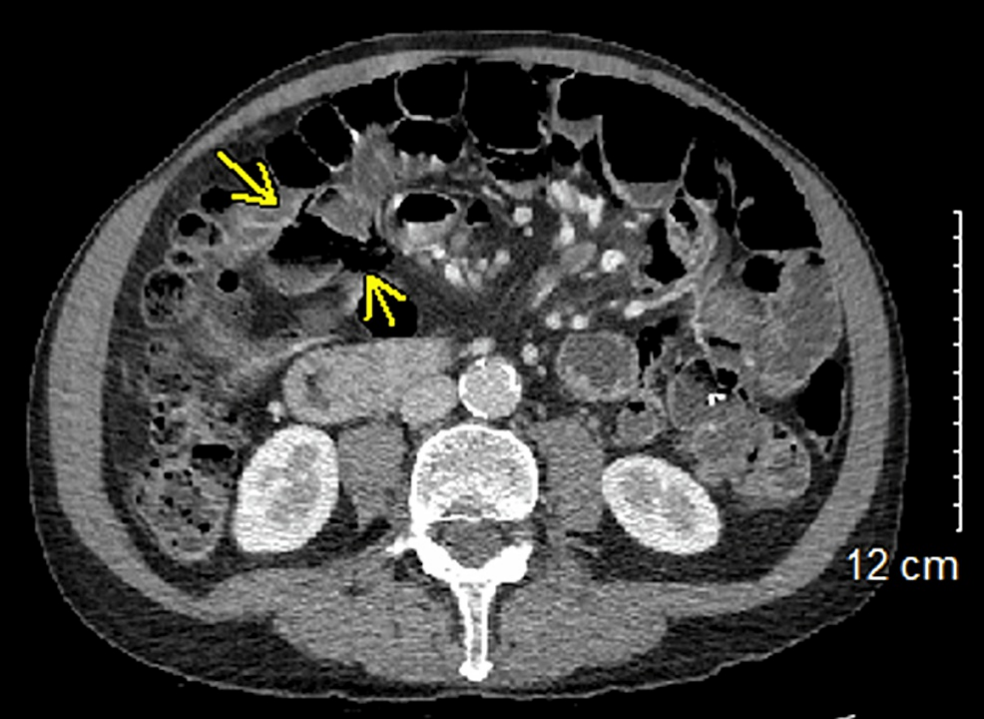
Axial contrast-enhanced abdominal CT showing an abnormal loop of small bowel in RUQ (yellow arrows) with diffuse pneumatosis CT: computed tomography; RUQ: right upper quadrant

**Figure 2:**
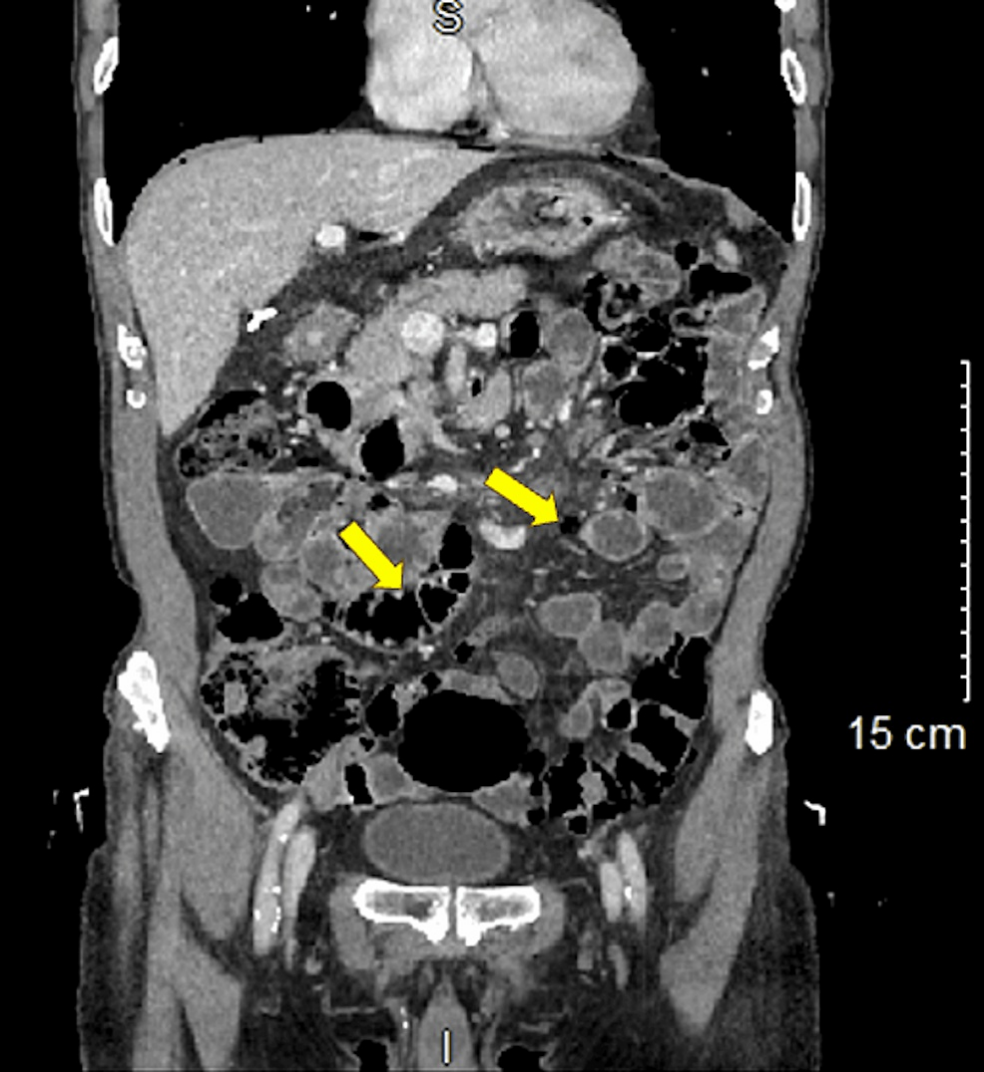
Coronal contrast-enhanced abdominal CT revealing pneumatosis cysts (yellow arrows) scattered throughout the small bowel

A 75-year-old Caucasian male presented to the emergency department with a two-day history of increasing constant diffuse generalized abdominal pain and dull back pain. His bowel function was normal, and he was tolerating oral intake. He denied nausea, vomiting, fever, melena, and hematochezia. His past medical history was significant for diverticulosis and irritable bowel syndrome with constipation (IBS-C), well-controlled with 290 mcg linaclotide once daily. The patient could not recall when he was diagnosed with diverticulosis or IBS-C, but he started experiencing gastrointestinal symptoms in early adulthood. Surgical history included endoscopic retrograde cholangiopancreatography (ERCP) with biliary stent placement and stent removal for choledocholithiasis. ERCP was followed by laparoscopic cholecystectomy, which occurred four months prior to this presentation. There was no history of a colonoscopy. He denied past and present tobacco use, and his family history was negative for colon cancer and gastrointestinal disease. His blood pressure was 182/95 mmHg, pulse was 88 bpm, respiratory rate was 16, and temperature was 36.4 °C. A physical exam revealed diffuse abdominal tenderness to palpation and voluntary guarding of the left umbilical region. A comprehensive metabolic panel and complete blood count in the emergency department showed no abnormalities. CT scan of the abdomen and pelvis with intravenous contrast revealed a moderate amount of free intraperitoneal air suggesting perforated viscus, likely related to an abnormal loop of small bowel in the right upper quadrant with possible pneumatosis (Figures 1-2). Sigmoid diverticulosis without evidence of diverticulitis was noted. The patient’s clinical presentation, age, history of gastrointestinal symptoms, and radiological evidence indicated the need for exploratory laparotomy to rule out bowel perforation.

**Figure 3:**
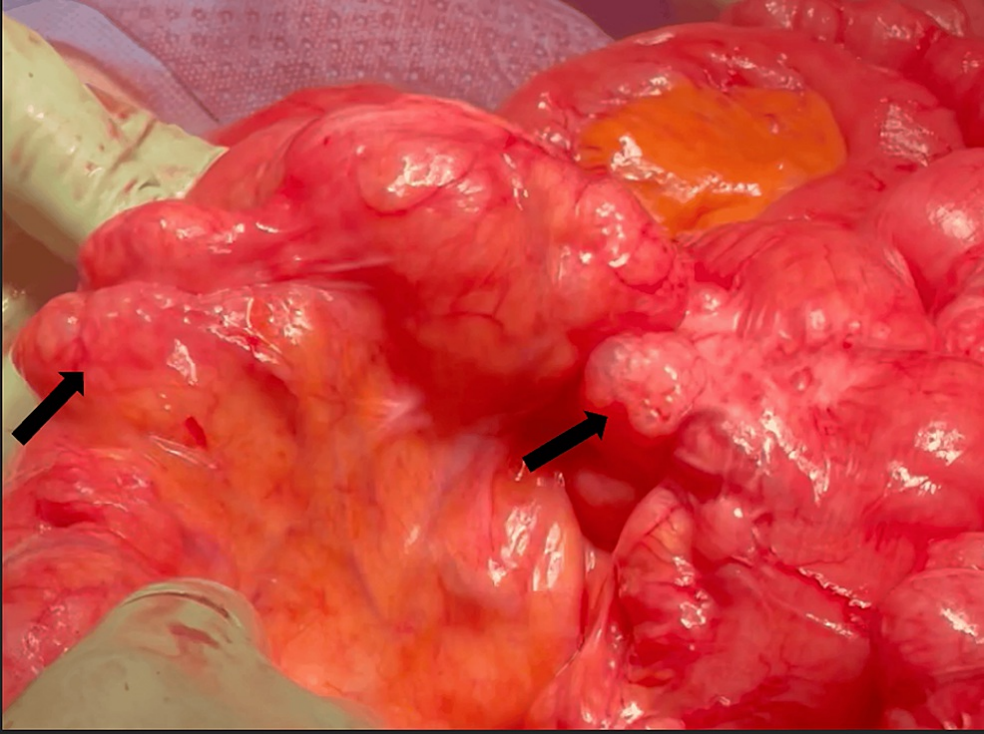
Small bowel with bubble-like cysts (black arrows) on the external surface

**Figure 4:**
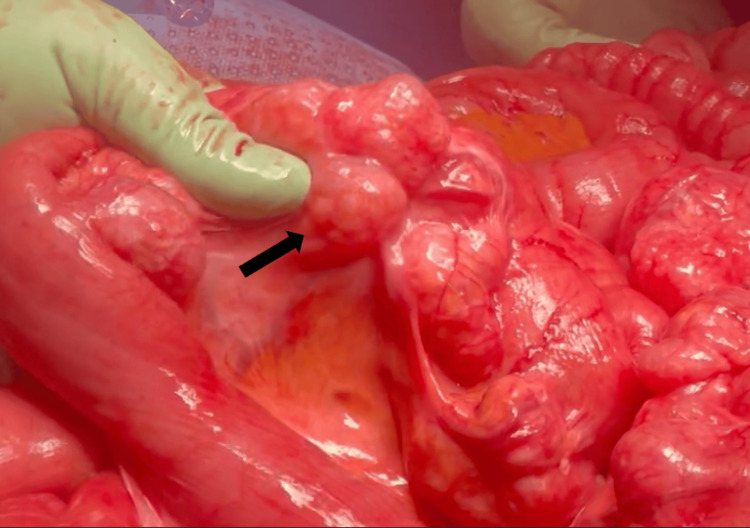
Small bowel with bubble-like cysts (black arrow) on the external surface

A 4.5 gm piperacillin-tazobactam intravenous infusion was administered, and the patient was transferred to the operating room for an exploratory laparotomy under general anesthesia. Upon exploration, intestinal malrotation in the right upper quadrant was present (Figure 1). Manual detorsion of this abnormal small bowel loop was performed without difficulty. The small bowel was run from the ligament of Treitz to the terminal ileum with no evidence of bowel perforation. Beginning at the ligament of Treitz through the jejunum, outpouchings filled with small air pockets were found on the mesenteric and anti-mesenteric small bowel surfaces (Figures 3-4). They decreased in size as we moved distally and terminated at the distal jejunum. The small bowel appearance was consistent with PCI. Examination of the large intestine revealed no abnormalities. Two lymph nodes were removed near a portion of the inflamed small bowel, and a biopsy revealed benign reactive lymph nodes. The abdomen was closed with one looped polydioxanone suture and staples. The patient tolerated surgery well and was admitted for postoperative observation. His abdominal and back pain resolved after surgery, and he tolerated oral intake on postoperative day 1. He was discharged on postoperative day 3 and was instructed to resume taking 290 mcg linaclotide once daily to treat IBS-C.

## Discussion

Current evidence suggests that intraluminal pressure, bacterial flora, and mucosal integrity play an integral role in PCI development [5]. Although no clear pathogenesis of PCI has been identified, there are several hypotheses. The mechanical theory attributes PCI development to mucosal damage caused by an increase in intraluminal pressure, a common result of abdominal surgery or trauma. The pulmonary theory hypothesizes that various lung diseases create alveolar destruction, causing free gas to enter the peritoneal cavity through the bloodstream. The bacterial theory correlates gas-producing bacteria to PCI development, and common bacterial culprits include *Clostridia* and *Escherichia* species [4]. PCI develops as a primary or secondary type. Primary PCI is typically benign, and a cystic pattern is seen diffusely in the intraluminal wall. Secondary PCI is associated with other pathological conditions, and the cystic pattern tends to be linear. Primary PCI is most often found in the small intestine, while secondary PCI is found in the ascending colon, but these patterns are not absolute [4,6]. Intestinal tract diseases correlated with PCI include diverticulosis, Crohn’s disease, ulcerative colitis, and intestinal stenosis [3]. We hypothesize that our patient’s history of IBS-C, diverticulosis, and previous abdominal surgery may have contributed to the development of PCI, but it is unclear if he had longstanding primary PCI or recently developed secondary PCI.

The prevalence of PCI is estimated to be 0.03% of the population, with increased prevalence in the elderly population. The nonspecific abdominal symptoms caused by PCI make it difficult to detect, and most cases remain asymptomatic [4]. Our patient’s symptoms in the emergency department were limited to abdominal pain and back pain, which may have been caused by intestinal malrotation as opposed to PCI (Figure 1). It is important to consider past medical history, surgical history, family history, tobacco use, and medication use. Our patient was a non-smoker with a non-contributory family history. He did have a history of IBS-C and diverticulosis, and he takes linaclotide daily. Prescription medications have been proven to contribute to PCI but more research must be done to identify which medications pose significant risk [3].

Contrast-enhanced abdominal CT is the current standard diagnostic imaging in PCI [2,3]. Modern imaging modalities have caused the prevalence of PCI to spike, and its unique appearance on abdominal CT is often mistaken for potential surgical emergencies. This increase in PCI prevalence is likely due to an increased number of confirmed cases that have remained undiagnosed in the past. Spreading awareness of PCI and the potential complications that may arise will provide a better framework for management to the medical community. Most cases of PCI remain benign but complications including bowel perforation, bowel ischemia, peritonitis, cystic rupture, and pneumoperitoneum can arise. Conservative management of PCI is always preferred, but if there is significant evidence of complications on contrast-enhanced abdominal CT, surgical intervention is necessary. Non-surgical management focuses on treating underlying pathologies that may be contributing to PCI development [5,7].

## Conclusions

The lack of understanding of PCI within the medical community often leads to confusion regarding management. Modern imaging modalities have led to an increased capability of identifying PCI, making it imperative to explore proper diagnosis and treatment. PCI has guideline algorithms that can assist in management, but no evidence-based guidelines exist for trauma scenarios. This can lead to misdiagnosis and misguided treatment. Once PCI is diagnosed, it is important to distinguish benign cases from potential surgical emergencies. Conservative treatment is always preferred in PCI, with the exception of complications that require surgical intervention.
